# Comparative transcriptomics and computational drug discovery identify ASPM as a key oncogenic driver and therapeutic target in hepatocellular carcinoma

**DOI:** 10.3389/fbinf.2026.1795889

**Published:** 2026-05-07

**Authors:** Pan Li, Aiye Guo, MingJing Zhao, GuangHui Chen

**Affiliations:** Department of Clinical Laboratory, Henan Provincial People’s Hospital, Zhengzhou University, Zhengzhou, Henan, China

**Keywords:** ASPM, calponin domain, comparative transcriptomics, fragment based drugs, HCC

## Abstract

**Introduction:**

Hepatocellular carcinoma (HCC) is a highly heterogeneous malignancy that necessitates the identification of robust biomarkers across diverse populations to enhance diagnostic and prognostic precision. This study aimed to identify clinically relevant biomarkers and potential therapeutic targets through integrative transcriptomic and computational analyses.

**Methods:**

A comparative transcriptomic analysis was performed on 724 HCC samples obtained from four independent cohorts (United States, South Korea, France, and Taiwan). Differential expression and survival analyses, including Kaplan–Meier estimation, were conducted to evaluate clinical significance. Functional enrichment analysis was used to explore biological roles. Structural modeling, molecular docking, 100-ns molecular dynamics (MD) simulations, MM-GBSA binding energy calculations, and *in silico* ADMET profiling were employed to assess ligand–target interactions.

**Results:**

Abnormal Spindle Microtubule Assembly (ASPM) was consistently overexpressed across all cohorts and significantly associated with poor overall survival. Functional analyses indicated its involvement in mitotic spindle organization and homologous recombination–mediated DNA repair. Among screened compounds, Mol-7424 exhibited stable binding within the ASPM calponin domain, favorable binding free energy, and promising pharmacokinetic properties. Lipid bilayer simulations further supported its membrane permeability and potential cellular uptake.

**Discussion:**

These findings highlight ASPM as a prognostic biomarker and potential therapeutic target in HCC. Mol-7424 emerges as a promising lead compound; however, its efficacy requires validation through *in vitro* and *in vivo* studies. Overall, this study underscores the utility of multi-population transcriptomics integrated with computational approaches for advancing precision oncology in HCC.

## 1 Introduction

Hepatocellular carcinoma (HCC) persists as a leading cause of cancer-related mortality worldwide, with shifting etiological trends and evolving therapeutic paradigms (Wu et al., 2021). While historically linked to viral hepatitis and chronic alcohol use, the rising prevalence of metabolic disorders, particularly non-alcoholic fatty liver disease (NAFLD), has reshaped HCC risk profiles, necessitating updated diagnostic and management strategies (Vogel et al., 2022; Wu et al., 2021). Current treatment modalities, including liver transplantation, surgical resection, and locoregional therapies, are guided by tumor stage and hepatic reserve, yet high recurrence rates and therapeutic resistance in advanced disease remain critical barriers to improving survival (Ganesan and Kulik, 2023). Although advances in systemic therapies, such as immune checkpoint inhibitors (e.g., atezolizumab-bevacizumab), have extended patient survival, disparities in global treatment protocols persist. For instance, Asian guidelines often favor more aggressive interventions than Western approaches, underscoring the need for harmonized, patient-tailored frameworks (Becht et al., 2024; Pathak and Sonbol, 2021).

Innovations in diagnostics, including radiomics and molecular profiling, offer non-invasive tools to refine prognostication and personalize care (Fang et al., 2023; Sun et al., 2023). However, the clinical translation of these tools is hindered by HCC’s molecular heterogeneity and a lack of robust biomarkers. Furthermore, racial disparities in treatment responses and limited inclusion in clinical trials highlight the urgency for equitable research practices (Garcia and Mathew, 2024). To address these challenges, bioinformatics and computational biology have emerged as transformative disciplines, enabling high-throughput analysis of multi-omics data to uncover conserved molecular signatures across diverse cohorts. Transcriptomic profiling, in particular, has accelerated the identification of differentially expressed genes (DEGs) with diagnostic and therapeutic potential, mitigating biases inherent to single-study datasets.

In the current study, we utilized an integrative bioinformatics approach to identify novel HCC biomarkers and therapeutic targets by analyzing four independent Gene Expression Omnibus (GEO) datasets (GSE22058, GSE62232, GSE87630, GSE101685) from four diverse populations (United States, South Korea, Taiwan, and France). By combining differential expression analysis, protein-protein interaction (PPI) networks, and pathway enrichment, we pinpoint upregulated hub genes consistently, with ASPM emerging as a top candidate. Recognizing the limitations of traditional drug development, we further employ *in silico* strategies—molecular docking and molecular dynamics (MD) simulations—to evaluate ASPM’s druggability. These computational techniques provide critical insights into ligand binding stability, affinity, and dynamic interactions, offering a rational framework for prioritizing therapeutic compounds. Our findings not only advance the quest for reliable HCC biomarkers but also bridge the gap between genomic discovery and actionable drug design, addressing unmet needs in precision oncology.

## 2 Materials and methods

### 2.1 Identification of DEGs from microarray datasets

To confirm the current study’s findings, four microarray datasets (GSE22058, GSE62232, GSE101685, and GSE87630) were retrieved from the Gene Expression Omnibus (GEO) database (Supplementary Table S1). Later, an R language program called “Limma” was used to look for differentially expressed genes (DEGs). Limma (Ritchie et al., 2015), which offers advanced features for reading, normalizing, and analyzing gene expression data, has emerged as the best option for identifying DEGs through differential expression analysis of high-throughput data and microarray data. Screening criteria were set to adj. p-value < 0.01. ggplot2 package (Gómez-Rubio, 2017). The ggplot2 package in R was used to construct a volcano plot to visualize important and non-significant genes. DEGs were then compared with hub genes, and genes with characteristics shared by both were selected for further investigation. Using the ggplot2 package, we generated a Venn diagram (Gao et al., 2021).

### 2.2 Comprehensive GO and KEGG pathway enrichment analysis

To determine the fundamental mechanism and pathways for all potential targets, we used Gene Ontology (GO) and Kyoto Encyclopaedia of Genes and Genomes (KEGG) pathway enrichment analysis (Aleksander et al., 2023; Xing et al., 2016). Functional annotation and pathway analysis were carried out using the Database for Annotation, Visualization, and Integrated Discovery (DAVID) (https://david.ncifcrf.gov/), an open-access functional enrichment database (Dennis et al., 2003). The Venn diagram’s common targets were submitted to DAVID, and “*Homo sapiens*” was selected as the species to predict gene functions at three levels: molecular function (MF), biological process (BP), cellular component (CC), and KEGG pathways. The KEGG pathway analysis allows us to better understand the biological system’s high-level functions and usefulness. The study employed an adjusted p-value of ≤ 0.05 to choose the top 10 GO enrichment and KEGG pathways with the highest counts for further examination. Following that, ggplot2 (Gómez-Rubio, 2017), an R tool, was used to depict critical GO terms and KEGG pathways.

### 2.3 Identification of hub genes through PPI network analysis

The Protein-Protein Interaction (PPI) network of target genes was created using the STRING database (https://string-db.org) with “*H. sapiens*” as the reference organism. Genes with a total score ≥0.5 were selected for further analysis, and the network was imported into Cytoscape 3.9.1 (Otasek et al., 2019). The STRING database is a free database for PPIs. It provides information on all known species, including proteins and interactions. The PPI network was collected from the STRING database and loaded into Cytoscape v.3.9.1 for identification. The network was then put into Cytoscape v.3.9.1, and the CytoHubba (Chin et al., 2014) plugin was applied. The CytoHubba degree-scoring algorithms were used to determine hub genes.

### 2.4 Survival analysis of ASPM in liver cancer

Gene expression and clinical data were obtained from The Cancer Genome Atlas Liver Hepatocellular Carcinoma (TCGA-LIHC) project via the Genomic Data Commons (GDC) portal using the TCGAbiolinks R package. RNA sequencing data were retrieved as STAR-aligned raw counts for Primary Tumor samples, and clinical metadata, including overall survival time, vital status, age at diagnosis, gender, tumor grade, and AJCC pathologic stage, were extracted from associated clinical files. ASPM (Abnormal Spindle-like Microcephaly Associated) gene expression was extracted. Samples were stratified into high and low ASPM expression groups using an optimal cutoff (log2 = 9.649) determined by the maximally selected rank statistics method (surv_cutpoint function, survminer R package). A total of 366 primary tumor samples with complete survival data were included for survival analysis. Overall survival (OS) was defined as the time from initial diagnosis to death from any cause, with censored data for patients without events. Kaplan-Meier survival curves were generated for high and low ASPM expression groups, and survival differences were evaluated using the log-rank test. Univariate Cox regression assessed the association between continuous log2-transformed ASPM expression and OS, and multivariate Cox regression evaluated ASPM’s independent prognostic value, adjusting for age at diagnosis and tumor stage. Tumor stage was simplified into two categories (Early: Stage I–II; Late: Stage III–IV) to address sparse subcategories causing model convergence issues. Gender and tumor grade were excluded due to non-significance and inferior AIC. Statistical significance was set at p < 0.05, and all analyses were conducted in R using the survival, survminer, and TCGAbiolinks packages.

### 2.5 Structural prediction using homology modeling

The three-dimensional structure of ASPM (UniProtKB: Q8IZT6) was predicted using homology modeling. This computational method constructs an atomic-resolution model of a protein from its amino acid sequence based on a homologous template structure. For modeling, the template A0A5N4CIN7.1.A, an abnormal spindle-like microcephaly-associated protein-like protein, was selected. The template exhibits 84.46% sequence identity with human ASPM and provides full coverage of the target sequence. The amino acid sequence of ASPM from residue 920-1318 encodes a highly conserved calponin domain. MODELLER (v10.5) (Webb and Sali, 2016) was utilized and following steps were performed 1) Template identification 2) Sequence alignment 3) Model building 4) model refinement 5) Model validation was performed to evaluate the structural quality of the refined ASPM model. This included assessment of backbone dihedral angles (φ, ψ) using Ramachandran plot analysis, evaluation of stereochemistry and side-chain conformations through MolProbity (calculating MolProbity score, clash score, rotamer outliers, C-Beta deviations, and geometric bond/angle quality), assessment of statistical potential energy via the DOPE score, comparison with non-redundant protein structures using QMEAN, and analysis of non-bonded atomic interactions using ERRAT.

### 2.6 Database selection and ligand preparation

Drug datasets were retrieved from Selleck’s Drug database (https://www.selleckchem.com). Selected 2,500 compounds were prepared, the energy was minimized, and the bond angle was optimized by default of the Universal Force Field (UFF) for each ligand using PyRx software (Dallakyan and Olson, 2015).

### 2.7 *In-silico* high-throughput screening

High-throughput screening (HTS) is a method used to identify new drug discovery leads by *in vitro* testing an extensive chemical library against a specific pharmacological target, cell, or organism. By prioritizing compounds for additional experimental inquiry, virtual screening (VS), a computer-aided method to imitate HTS, can speed up the drug discovery process and cut expenses while lowering the failure rate. We performed a targeted screening following energy minimization of selected hits. This screening against the ASPM calponin domain was performed with AutoDock Vina (Eberhardt et al., 2021), which was assembled in PyRx (Dallakyan and Olson, 2015).

### 2.8 Molecular docking

Docking analysis involves evaluating the score function used, interpreting the docking score values, and assessing ligand interactions. The PyRx (Dallakyan and Olson, 2015) software was utilized for targeted docking. A blind docking approach was utilized following the previously reported method (Arain et al., 2024; Safdar et al., 2024). The docking simulation was then run with an exhaustiveness of 8 and set to output only the lowest-energy pose. Using the BIOVA Discovery Studio Visualizer Tool 2021 (Shaweta, Akhil, and Utsav, 2021), the docked chemical that had the best binding affinity (kcal/mol) was fetched and visualized.

### 2.9 Molecular dynamics simulation

All-atom molecular dynamics (MD) simulations were performed using GROMACS version 2024.1 (Abraham et al., 2015). The CHARMM36-jul2021 force field was applied for the protein, while ligand parameters were generated using the CHARMM General Force Field (CGenFF v4.6) (Huang and Mackerell, 2013) through the CGenFF webserver. The protein–ligand complex was placed in a triclinic simulation box with a minimum distance of 1.2 nm from the box edges and solvated using the TIP3P (Mark and Nilsson, 2001) water model. Na^+^ and Cl^−^ ions were added to neutralize the system and maintain a physiological salt concentration of 0.15 M. Energy minimization was carried out using the steepest descent algorithm until the maximum force was below 500 kJ/mol/nm (Darden et al., 1993). The system was equilibrated under NVT (300 K) and NPT (1 bar) ensembles using the V-rescale thermostat (Bussi et al., 2007) and Parrinello–Rahman barostat, respectively. Long-range electrostatic interactions were calculated using the Particle Mesh Ewald (PME) method with a cutoff of 1.2 nm, and periodic boundary conditions were applied in all directions. Hydrogen bonds were constrained using the LINCS algorithm (Hess et al., 1997). Production MD simulations were performed for three independent replicates of 100 ns each with a 2 fs time step (Páll and Hess, 2013). Structural stability and conformational dynamics were evaluated by calculating root mean square deviation (RMSD), root mean square fluctuation (RMSF), and radius of gyration (Rg). Protein–ligand interaction energies were also calculated, and free energy landscape (FEL) analysis was performed to identify the dominant conformational states sampled during the simulation.

### 2.10 Binding free energy calculation, MM/GBSA

Binding free energies of the docked ligands with the ASPM calponin domain were estimated using the MM-GBSA approach as implemented in Maestro’s Prime module (Bell et al., 2012). To ensure reliable results, the most favorable docking poses were first selected, and both receptor and ligands were preprocessed using Protein Preparation Wizard and LigPrep to optimize geometries, assign protonation states, and refine hydrogen bonding networks. MM-GBSA calculations were performed on the optimized complex, the free receptor, and the free ligand. Snapshots for the calculations were extracted from the last 50 ns of the 100 ns MD trajectories at 10 ps intervals to sample representative conformations. Entropic contributions (TΔS) were not included due to computational constraints; therefore, the results provide estimates of relative binding affinities rather than absolute free energies. Table 1 summarizes the binding energy components, including electrostatic (ΔE_col), bonded (ΔE_cov), solvation (ΔE_sol), hydrogen bonding (ΔE_hb), van der Waals (ΔE_vdw), lipophilic (ΔE_lipo), packing (ΔE_Packing), and total contributions (ΔE_tol and ΔE_com).

**TABLE 1:** Estimation of free binding energy using MMGBSA analysis.

Complex	Polar				Net polar	Non-polar			Net non-polar	Total	
	ΔE_col_	ΔE_cov_	ΔE_sol_	ΔE_hb_		ΔE_vdw_	ΔE_lipo_	ΔE_Packing_		ΔE_tol_	ΔE_com_
Mol-7424	13.21	4.21	1.10	−2.04	16.48	−19.03	−4.92	−1.05	−25.00	−8.52	−16742.89
Mol-273	4.75	0.85	15.55	−0.24	20.92	−22.45	−3.50	0.00	−25.95	−5.03	−16730.74
Mol-7112	−7.47	0.39	18.90	−0.75	11.08	−21.59	−11.1	−1.48	−34.24	−23.16	−16718.43

### 2.11 Free energy landscape (FEL)

Free Energy Landscape (FEL) analysis was performed to assess protein-ligand interactions and conformational stability throughout molecular dynamics (MD) simulations (Al-Khafaji and Tok, 2020). Covariance analysis was conducted in GROMACS, where gmx covar computed atomic motion correlations, generating eigenvalues.xvg and eigenvectors.trr files. Principal component analysis (PCA) was performed using gmx anaeig, with PC1 and PC2 accounting for the most significant conformational variations. The FEL was mapped onto a two-dimensional energy surface using gmx sham and visualized with Python-based Matplotlib and NumPy. The five lowest-energy minima were identified, capturing critical stability parameters such as RMSD, RMSF, hydrogen bonding interactions, and eigenvalues, offering deeper insights into the dynamic behavior and binding stability of the ligand-protein complex (Al-Khafaji and Tok, 2020).

### 2.12 *In silico* pharmacokinetics and toxicity evaluation

Pharmacokinetic and drug-likeness properties of Mol-7424 and comparator molecules (Mol-7112 and Mol-273) were evaluated using two well-established web-based platforms: SwissADME (http://www.swissadme.ch/) and ADMETlab 2.0 (https://admetmesh.scbdd.com/). Molecular descriptors such as molecular weight (MW), hydrogen bond acceptors (HBA), hydrogen bond donors (HBD), topological polar surface area (TPSA), lipophilicity (WLOGP), and Lipinski’s Rule of Five compliance were computed via SwissADME. The BOILED-Egg model was used to predict passive gastrointestinal (GI) absorption and blood-brain barrier (BBB) permeability, along with P-glycoprotein (P-gp) substrate status to assess potential efflux liabilities.

For ADMET-related properties, bioavailability scores, plasma protein binding (PPB), and clearance rate (CLplasma) were retrieved from ADMETlab 2.0. These metrics allowed evaluation of systemic exposure potential, elimination profile, and tissue selectivity. All computational predictions were performed using the compounds’ canonical SMILES, with default settings enabled on each platform.

### 2.13 Advanced lipid bilayer dynamics simulations

This lipid bilayer model was set up at CHARMM-GUI with POPC (1-Palmitoyl-2-Oleoyl-sn-Glycero-3-Phosphocholine), POPE (1-Palmitoyl-2-Oleoyl-sn-Glycero-3-Phosphoethanolamine), and cholesterol (CHL1) in a 64:32:32 ratio for both leaflets. The system is solvated with TIP3P water and neutralized by potassium (POT), sodium (SOD), and chloride (CLA) ions to maintain physiological conditions. Energy minimization and equilibration were performed using GROMACS under a stepwise protocol, including steepest-descent minimization, NVT ensemble equilibration, and NPT ensemble equilibration with positional restraints. Production Molecular Dynamics (MD) simulations were performed for 100 ns under periodic boundary conditions with Particle Mesh Ewald (PME) electrostatics, LINCS constraints for bonds, and a timestep of 2 fs. Post-simulation analyses included solvent-accessible surface area (SASA), root-mean-square deviation (RMSD), penetration depth, drug-target distance, free energy landscape, radial distribution function (RDF), and ligand density calculations.

## 3 Results

### 3.1 Comparative analysis of transcriptomes across diverse populations

**FIGURE 1:**
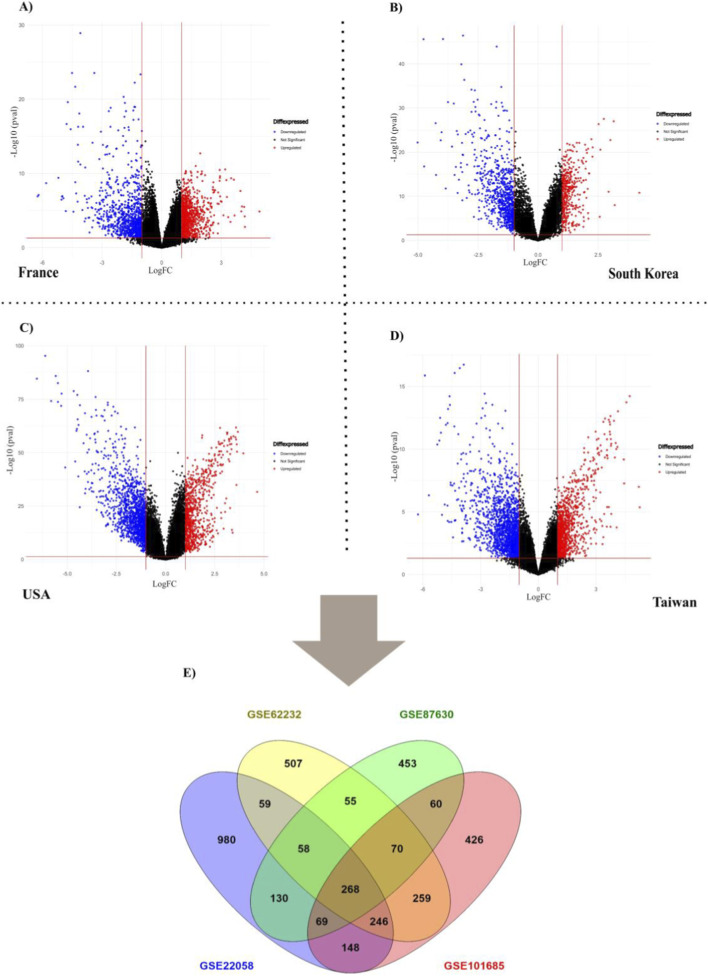
Transcriptome comparison of four diverse datasets. **(A–D)** Volcano plots showing differentially expressed genes (DEGs) between OSCC and normal tissues in four independent datasets: GSE22058 (United States), GSE62232 (France), GSE101685 (Taiwan), and GSE87630 (South Korea). DEGs were identified using GEO2R, which applies the limma package internally, and results were confirmed with limma in R. Genes with an adjusted p-value < 0.05 and |log_2_ fold-change| > 1.0 were considered significant. Volcano plots were generated using the ggplot2 package in R. **(E)** Venn diagram showing overlapping DEGs across the four datasets, generated with Venny 2.1.A total of 268 common genes were identified across all four datasets, highlighting shared transcriptomic alterations.

Transcriptome datasets from multiple regions, including the United States, South Korea, France, and Taiwan, were retrieved from the GEO expression database (Supplementary Table S1). Differentially expressed genes (DEGs) were identified in affected liver tissues using the R package Limma with a custom R script, considering normal tissues as controls. Genes were classified as DEGs based on a stringent threshold of an adjusted p-value < 0.01 (Table 2). In GSE22058 (United States), 801 upregulated and 1,461 downregulated genes were identified. Similarly, GSE62232 (France) exhibited 1,130 upregulated and 769 downregulated genes (Figure 1). GSE87630 (South Korea) revealed 395 upregulated and 769 downregulated genes, while GSE101685 (Taiwan) had 1,179 upregulated and 1,902 downregulated genes. A comparative analysis of these datasets identified 268 common DEGs across all four populations, comprising 64 upregulated and 204 downregulated genes. To visualize the overlap, a Venn diagram was constructed to illustrate the shared and unique gene sets across the datasets (Figure 1).

**TABLE 2:** Diverse datasets utilized for HCC transcriptome comparison.

Sr.	Dataset	Platform	Control	Affected	Total	Upregulated	Downregulated	Tissue	Region
1	GSE22058	GPL6793	97	100	197	801	1461	Liver	USA
2	GSE87630	GPL6947	30	64	94	395	769	Liver	South Korea
3	GSE62232	GPL570	10	81	91	1130	769	Liver	France
4	GSE101685	GPL570	8	24	32	1179	1902	Liver	Taiwan

### 3.2 Functional annotation of commonly expressed genes

Gene Ontology (GO) and Kyoto Encyclopedia of Genes and Genomes (KEGG) pathway analyses were performed using the Database for Annotation, Visualization, and Integrated Discovery (DAVID) to elucidate the functional roles of differentially expressed genes (DEGs) in hepatocellular carcinoma (HCC). DEGs were systematically categorized into three GO domains: biological processes, molecular functions, and cellular components, revealing their distinct contributions to HCC pathophysiology (Supplementary Figure S1). Biological process analysis highlighted significant enrichment in metabolic and catabolic pathways, including *drug metabolism*, *steroid metabolic processes*, and *organic acid catabolism*. Notably, the epoxygenase P450 pathway emerged as a key player, underscoring the critical role of metabolic reprogramming in HCC progression. These findings align with the tumor’s reliance on altered metabolic pathways to sustain proliferation and evade therapeutic interventions. Within the molecular function domain, DEGs were strongly associated with enzymatic and binding activities governing redox balance and biosynthesis. Enriched terms such as *steroid hydroxylase activity*, *monooxygenase activity*, and *heme/iron ion binding* implicate oxidative stress regulation and xenobiotic detoxification as central mechanisms in HCC development. The prominence of *arachidonic acid epoxygenase activity* further suggests dysregulated lipid signaling in tumorigenesis. Cellular component analysis revealed enrichment in structures linked to extracellular matrix remodeling and lipid metabolism. Key terms included the *MCM complex* (implicated in DNA replication), *blood microparticles*, *plasma lipoprotein particles*, and *collagen-containing extracellular matrix*. These findings reflect the profound extracellular matrix reorganization and lipid dysregulation characteristic of HCC’s tumor microenvironment. KEGG pathway analysis identified critical pathways driving HCC progression, including *drug metabolism via cytochrome P450*, *chemical carcinogenesis (DNA adduct formation)*, *fatty acid degradation*, and *pyruvate metabolism*. The convergence of these pathways highlights the interplay between genetic alterations, metabolic dysfunction, and carcinogen-induced DNA damage in HCC. For instance, dysregulated *complement and coagulation cascades* may promote tumor immune evasion, while aberrant *DNA replication* pathways align with genomic instability in HCC.

### 3.3 Identification of potential biomarkers via protein-protein interaction (PPI) network analysis

**FIGURE 2:**
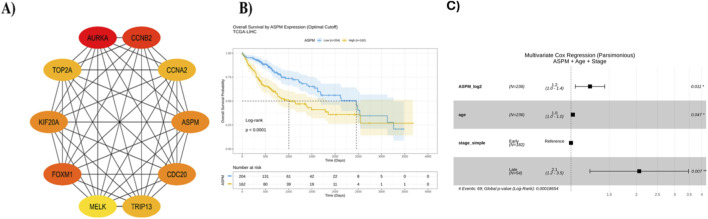
Protein-protein interaction (PPI) **(A)** The top 10 hub genes identified using the CytoHubba plugin in Cytoscape based on the Degree algorithm. **(B)** Kaplan–Meier overall survival analysis of TCGA-LIHC patients (n = 366) stratified by ASPM expression (optimal cutoff log_2_ = 9.649). High ASPM expression (n = 162) was associated with significantly poorer survival compared to low expression (n = 204) (median OS: 1,005 vs. 2,456 days; log-rank *p* < 0.0001). Shaded areas indicate 95% confidence intervals. **(C)** Multivariate Cox regression analysis showing that ASPM expression is an independent predictor of poor overall survival, after adjustment for age and tumor stage (HR = 1.223, 95% CI: 1.047–1.430, *p* = 0.011). Squares represent hazard ratios and horizontal lines indicate 95% confidence intervals.

To explore the functional interactions among differentially expressed genes (DEGs), the common genes identified across all datasets were uploaded to the STRING database. This analysis generated a comprehensive PPI network, where nodes represent proteins and edges indicate interactions between them (Supplementary Figure S2). A higher edge density suggests stronger functional correlations and greater biological significance of the associated proteins. Further examination of the PPI network revealed distinct clusters of functionally related proteins. Notably, genes involved in the cell cycle (e.g., CCNA2, FOXM1, AURKA, TOP2A, and ASPM) formed a highly interconnected module, emphasizing their collective role in cell proliferation and division, key processes in hepatocellular carcinoma (HCC) progression. Similarly, proteins involved in DNA replication and repair, such as MCM2, MCM4, and POLA1, showed strong interactions, underscoring their role in genomic stability and tumor cell survival. Additionally, metabolism-related proteins, particularly members of the cytochrome P450 family, showed significant interconnections within the network. These enzymes play crucial roles in drug metabolism and detoxification, underscoring their involvement in HCC progression and chemoresistance. Another distinct cluster comprised extracellular matrix (ECM) components and adhesion-related proteins, including COL1A1, COL3A1, and FN1, reflecting their role in extracellular remodeling, tumor invasiveness, and metastasis. To pinpoint key regulatory genes within the network, the PPI data from STRING were further analyzed using Cytoscape. Employing the CytoHubba plugin, we applied Degree scoring algorithms to identify key interaction hubs. Based on these analyses, ten hub genes were shortlisted for each metric, and five common genes, ASPM, FOXM1, AURKA, CCNA2, and TOP2A, were identified as critical nodes in the network (Figure 2A).

### 3.4 ASPM as a candidate novel biomarker for HCC

The PPI network (Figure 2A) shows the top 10 hub genes identified by CytoHubba in Cytoscape. These genes, including AURKA, CCNB2, CCNA2, FOXM1, ASPM, and others, form a highly interconnected network, suggesting their significant roles in hepatocellular carcinoma (HCC) progression. The color gradient from yellow to red represents the ranking of these genes by their connectivity, with AURKA, CCNB2, and ASPM showing the highest interaction density. Functionally, these genes are primarily involved in cell cycle regulation, mitotic progression, and tumor proliferation. Their strong interactions highlight their collective influence on cancer cell division and growth, reinforcing their potential as key regulatory elements in HCC pathogenesis.

### 3.5 Survival analysis of ASPM in liver cancer

A total of 366 primary tumor samples from TCGA-LIHC were included in the analysis, with 130 recorded death events (35.5%). Using the optimal ASPM expression cutoff (log2 = 9.649), samples were stratified into high (n = 162) and low (n = 204) ASPM expression groups. Kaplan-Meier survival analysis showed that patients with high ASPM expression had significantly shorter overall survival (OS) compared to those with low ASPM expression. The median OS for the high ASPM group was 1,005 days (95% CI: 757–1,852 days), compared with 2,456 days (95% CI: 1,685–3,258 days) for the low ASPM group (Figure 2B). The survival difference between these groups was statistically significant (log-rank test, p < 0.05), indicating that elevated ASPM expression is associated with poorer prognosis in HCC. Univariate Cox regression revealed that higher ASPM expression was significantly associated with an increased risk of death (HR = 1.233, 95% CI: 1.108–1.372, p = 0.0001), with a concordance index of 0.637, indicating moderate discriminative ability. In multivariate Cox regression, adjusting for age and tumor stage, ASPM expression remained a statistically significant independent predictor of OS (HR = 1.223, 95% CI: 1.047–1.430, p = 0.011). Age (HR = 1.020, 95% CI: 1.000–1.039, p = 0.047) and late tumor stage (HR = 2.061, 95% CI: 1.222–3.475, p = 0.007) were also significant predictors (Figure 2C). The overall multivariate model was highly significant (likelihood ratio test p = 0.0002, Wald test p = 0.0001), with a concordance index of 0.679, indicating good prognostic discrimination. These findings confirm that ASPM expression is an independent prognostic biomarker in hepatocellular carcinoma, with higher ASPM expression associated with significantly worse OS after adjusting for age and tumor stage.

### 3.6 Prediction of ASPM calponin domain structure

Identification of drug fragments requires extensive protein structural study. Since the ASPM structure was not available. We predicted conserved domain structure using Modeller. We selected the calponin domain of ASPM that is typically involved in binding to actin filaments and regulating cytoskeletal dynamics. The calponin domain of ASPM could be an ideal drug target due to its conserved structure, role in cell division, association with microcephaly, potential for specificity, and structural stability. We selected the ASPM amino acid region (920-1318) and used Modeler for structure prediction with a template. After homology modeling and refinement, the ASPM protein model was rigorously validated using multiple approaches. The backbone dihedral angles (φ and ψ) were evaluated using the Ramachandran plot (Supplementary Figures S3A,B), revealing that 93.2% (371/398) of residues were located in the favored regions and 99.2% (395/398) fell within allowed regions, which include less common conformations that do not introduce steric clashes. Only three residues, Ile183, Gly275, and Asn280, were identified as outliers, indicating that the predicted structure is stereochemically stable, and the small number of outliers is typical of high-quality protein models. Further assessment with MolProbity demonstrated a MolProbity score of 1.09, a clash score of 0.46, and only 0.82% rotamer outliers, reflecting excellent stereochemical quality and highly reliable side-chain conformations. Bond and angle geometry analysis revealed no bad bonds and only 22 out of 4,418 bad angles, while minor C-Beta deviations were observed at residues SER266, ASP267, and ASP106, representing acceptable local deviations. Together with DOPE, QMEAN, and ERRAT assessments, these results confirm that the refined ASPM model possesses high stereochemical and geometrical quality, making it suitable for downstream structural, functional, and molecular dynamics analyses (Supplementary Figure S3).

### 3.7 Virtual screening and molecular docking

Virtual screening using a blind molecular docking approach identified three candidate drug fragments: 1) Mol-7112 (4,4′-Dihydroxybiphenyl), 2) Mol-7424 (3,5-Dihydroxybenzoic acid), and 3) Mol-273 (1-BOC-3-piperidine carboxylic acid). Individual docking of these molecules to the ASPM calponin domain revealed distinct interaction profiles and binding affinities (Table 3; Supplementary Table S2). Mol-7112 exhibited the lowest docking energy (−6.3 kcal/mol), indicating the strongest predicted binding affinity. Mol-7424 and Mol-273 showed moderately lower binding affinities (−4.8 and −4.5 kcal/mol, respectively). Despite this, detailed interaction analysis revealed that Mol-7424 forms multiple conventional hydrogen bonds with LYS214 and ASP218, which are generally strong and contribute to the stability of the protein-ligand complex. Mol-273 also formed conventional hydrogen bonds with VAL231, whereas Mol-7112 lacked conventional hydrogen bonds but engaged in π–π stacking with PHE206 and π-anion interactions with ASP218 and LYS214, suggesting alternative stabilizing interactions. All three ligands participated in van der Waals interactions, ensuring shape complementarity with the ASPM calponin domain. π-Alkyl interactions were observed for Mol-7424 and Mol-7112 with LYS214, and for Mol-273 with ALA225. These collective interactions highlight the diverse modes through which each ligand can bind the target. While docking scores provide a preliminary quantitative assessment, they are inherently predictive. To further evaluate ligand stability and binding, molecular dynamics simulations were performed, revealing that Mol-7424 maintains sustained hydrogen bonding and minimal RMSD fluctuations, supporting its potential as a stable binder to the ASPM calponin domain. Future studies, including comparison with experimentally validated ligands or controls, will further strengthen the quantitative interpretability of these findings.

**TABLE 3:** Interaction of ASPM calponin domain with three candidate drug fragments.

Complex	Conventional H-bond	π – π stacked	Van der Waals interactions	π – alkyl bond	π – anion
Mol-7424 4,4′Dihydroxybiphenyl	LYS214, ASP218		GLU207, MET217, SER203, PHE206, ASP202	LYS214	------------
Mol-7112 3,5-Dihydroxybenzoic acid	------------	PHE206	ASN221, MET217, SER203, GLU207	LYS214	ASP218, LYS214
Mol-273 1-BOC-3-piperidine carboxylic acid	VAL231		ASP202, GLU232, ASN221, PHE206, ALA222, LYS230, LYS229	ALA225	------------

### 3.8 Molecular dynamics-driven exploration of ASPM and drug fragments binding mechanisms

**FIGURE 3:**
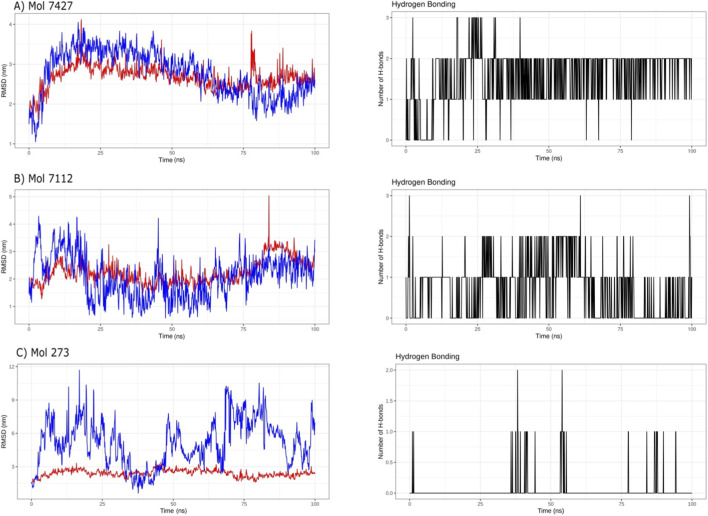
Molecular dynamics simulation analysis of ASPM calponin domain in complex with selected drug fragments over 100 ns. **(A)** RMSD trajectory and hydrogen-bond analysis of the ASPM–Mol-7424 complex showing relatively stable protein and ligand conformations with intermittent formation of up to three hydrogen bonds. **(B)** RMSD and hydrogen-bond profile of the ASPM–Mol-7112 complex, demonstrating moderate fluctuations in ligand stability and variable hydrogen-bond interactions. **(C)** RMSD and hydrogen-bond analysis of the ASPM–Mol-273 complex showing higher ligand mobility and fewer persistent hydrogen-bond interactions. The plots represent the average trajectories obtained from three independent MD simulations (triplicates).

To further investigate the atomistic interactions between the ASPM calponin domain and the selected drug fragments, 100 ns molecular dynamics (MD) simulations were performed in triplicate, and the averaged trajectories were analyzed. For Mol-7424, the protein RMSD remained relatively stable throughout the simulation, with moderate fluctuations around 2.0–3.5 Å, indicating that the overall protein structure remained stable across all replicates. The ligand RMSD also exhibited controlled fluctuations within a similar range, suggesting that Mol-7424 maintained a stable binding orientation within the ASPM binding pocket. Consistently, the hydrogen-bond analysis across the averaged trajectories revealed intermittent yet persistent interactions, with up to three hydrogen bonds observed during the simulation period (Figure 3A).

In the case of Mol-7112, the RMSD profiles showed greater fluctuations than those of Mol-7424. While the protein RMSD remained within an acceptable range, the ligand RMSD exhibited larger variations across the simulation, indicating moderate instability in ligand positioning within the binding pocket. Correspondingly, the hydrogen-bond analysis revealed fewer, less persistent interactions, with most simulations showing 0–2 hydrogen bonds (Figure 3B).

For Mol-273, the simulations demonstrated the greatest fluctuation, particularly in the ligand RMSD, which exhibited large deviations throughout the trajectory. Although the protein RMSD remained relatively stable, the ligand showed considerable mobility within the binding site, suggesting weaker binding. The hydrogen-bond analysis further supported this observation, showing only occasional and short-lived hydrogen-bond interactions, generally limited to 0–1 bonds throughout the simulation (Figure 3C).

Overall, the averaged results from the triplicate MD simulations indicate that Mol-7424 forms the most stable interaction with the ASPM calponin domain, as reflected by comparatively lower RMSD fluctuations and more consistent hydrogen-bond formation. In contrast, Mol-7112 and Mol-273 exhibit greater ligand mobility and fewer stable hydrogen-bond interactions, suggesting comparatively weaker binding stability within the ASPM binding pocket.

### 3.9 Evaluation of the molecular flexibility of the calponin domain upon interaction with drug fragments

**FIGURE 4:**
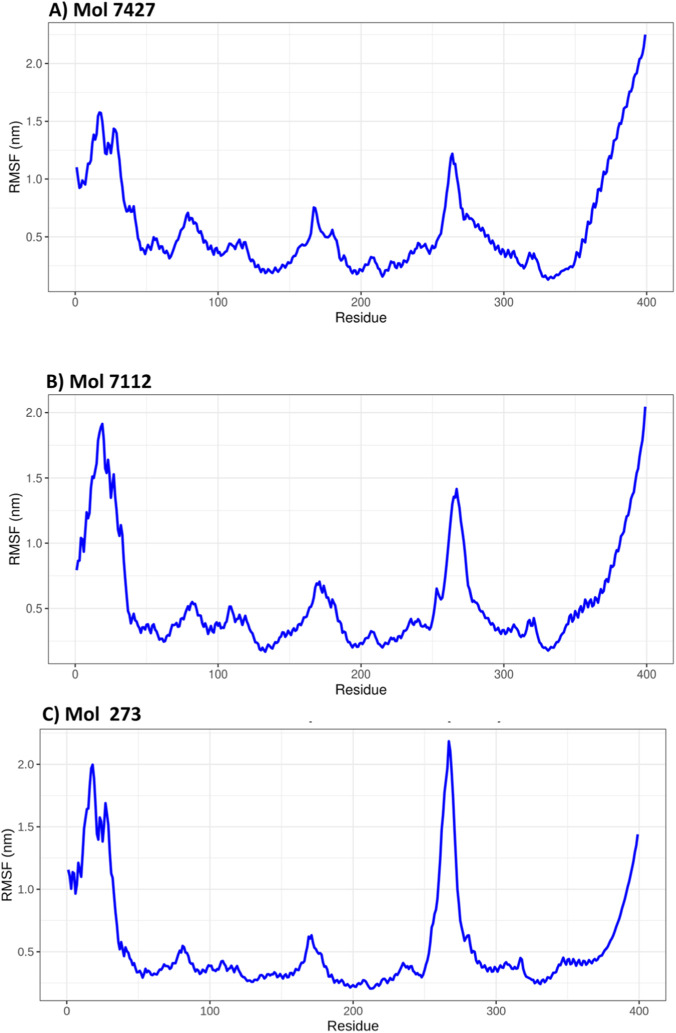
Root mean square fluctuation (RMSF) profiles of the ASPM calponin domain calculated from three independent 100 ns molecular dynamics simulations (triplicates). **(A–C)** Correspond to complexes with Mol-7424, Mol-7712, and Mol-273, respectively. The RMSF plots reveal regions of increased flexibility primarily in the N-terminal region (∼10–40 residues), around residues 160–190 and 250–270, and the C-terminal region (>360 residues). The central core of the protein remains comparatively stable across all complexes during the simulations. 3.4 Structural compactness of ASPM evaluation in a dynamic environment.

All three drug fragments induce flexibility in the ASPM calponin domain, but the specific regions and extent of this flexibility vary between the fragments. Common regions of increased flexibility are observed around residues 50–100 and 300–350 across all three interactions (Figures 4A–C). The unique peaks observed in the interactions with Mol-7112 (Figure 4C) and Mol-273 (Figure 4B), such as the peak around residues 250–300 for Mol-7112 (Figure 4C) and the sharp increase after residue 350 for Mol-273 (Figure 4B), suggest that these drug fragments may affect the ASPM calponin domain differently. All three drug fragments induce flexibility in the ASPM calponin domain, but the specific regions and extent of this flexibility vary between the fragments. Common regions of increased flexibility are observed around residues 50–100 and 300–350 across all three interactions. The differences in the RMSF profiles highlight the unique interactions of each drug fragment with the ASPM calponin domain.

To evaluate residue-level flexibility of the ASPM calponin domain upon ligand binding, root mean square fluctuation (RMSF) analysis was performed based on triplicate 100 ns molecular dynamics simulations for each protein–ligand complex (Figures 4A–C).

The RMSF profile indicates relatively low fluctuations for the majority of residues, generally ranging between 0.2 and 0.6 nm, suggesting stable backbone dynamics. Higher fluctuations are primarily observed in the N-terminal region (∼10–40 residues), reaching approximately 1.5 nm, which is typical for terminal segments. A moderate peak is also detected around residues ∼250–270, suggesting localized flexibility in this region. Additionally, the C-terminal region (>360 residues) shows a gradual increase in RMSF values, indicating increased mobility toward the end of the protein sequence (Figure 4A).

The RMSF profile of the Mol-7112 complex shows overall behavior comparable to that of the free protein, with most residues maintaining relatively low fluctuations. However, a distinct and sharp peak around residues ∼250–270 exceeds 2.0 nm, indicating increased local mobility in this region. The N-terminal segment (∼10–40 residues) also shows elevated fluctuations, while the C-terminal region (>360 residues) again exhibits a progressive increase in RMSF values (Figure 4B).

A broadly similar fluctuation pattern is observed for the Mol-273 complex, with most residues maintaining relatively low RMSF values. However, slightly higher fluctuations are observed in the N-terminal region (∼10–40 residues), reaching values close to 2.0 nm. Moderate flexibility is also evident around residues ∼160–190, while a noticeable peak occurs around residues ∼250–270. Similar to the previous complex, the C-terminal residues (>360) display a gradual increase in fluctuation values (Figure 4C).

Overall, the RMSF analysis across the three complexes indicates that ligand binding mainly induces localized flexibility in terminal and loop regions, whereas the central structural core of the ASPM calponin domain remains relatively stable throughout the simulations.

### 3.10 Structural compactness of ASPM in a dynamic environment

The radius of gyration (Rg) trajectories obtained from replicate molecular dynamics simulations were analyzed to evaluate the structural compactness of the ASPM–ligand complexes. Among the three systems, the ASPM–Mol-7427 complex showed an initial decrease in Rg followed by stabilization around ∼2.9 nm, suggesting gradual structural compaction and attainment of conformational equilibrium during the simulation.

In contrast, the ASPM–Mol-7112 complex exhibited larger fluctuations throughout the trajectory, with noticeable peaks and transient increases in Rg. This behavior indicates enhanced conformational flexibility of the protein upon interaction with Mol-7112, suggesting a less rigid binding mode.

**FIGURE 5:**
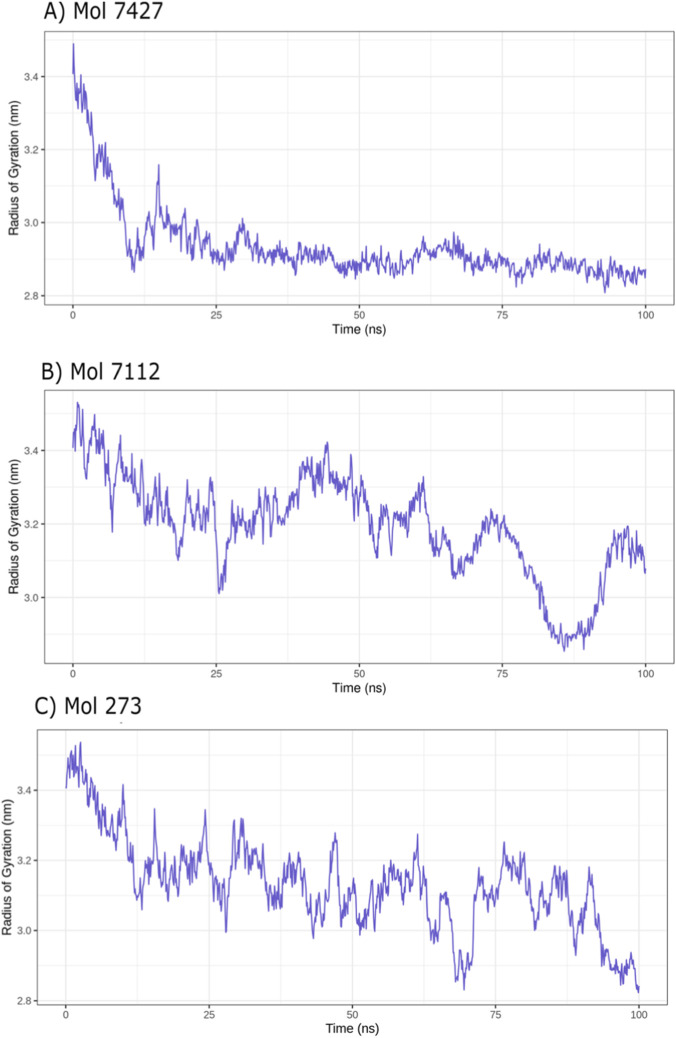
Radius of gyration (Rg) analysis of ASPM calponin domain upon interaction with drug fragments. Rg trajectories of the 400 amino acid calponin domain were monitored over 100 ns molecular dynamics simulations (triplicates) for complexes with **(A)** Mol-7424, **(B)** Mol-7112, and **(C)** Mol-237. The radius of gyration reflects the overall compactness of the protein structure, with lower values indicating a more compact conformation and higher values suggesting expansion or flexibility. Simulations were performed using GROMACS with the CHARMM36 force field under NPT ensemble conditions, 2 fs timestep, and periodic boundary conditions. Observed trends provide insights into structural stability and conformational changes induced by ligand binding.

Similarly, the ASPM–Mol-273 system demonstrated persistent variability in Rg values, although a general decreasing trend toward the end of the simulation suggests progressive structural adaptation of the protein. Overall, Mol-7427 maintained comparatively stable structural compactness, whereas Mol-7112 and Mol-273 induced higher dynamic fluctuations, implying differential effects of ligand binding on ASPM conformational stability (Figure 5).

### 3.11 MMGBSA analysis

We further estimated the binding affinity of the candidate drug fragment with the ASPM calponin domain (Table 1). Mol-7424, represent higher contribution H-bond energies however unfavorable polar interaction were also observed. Contrastingly, the non-polar interactions, particularly the van der Waals contributions, are significantly favorable, suggesting strong hydrophobic interactions with the domain. The total free binding energy is negative (−8.52 kcal/mol), indicating a stable interaction with the ASPM calponin domain. Mol-273 has shown relatively higher total free binding energy (−5.03 kcal/mol) indicating the stable binding affinity. Finally, the Mol-7112 shows the most stable interaction based on the total free binding energy. The favourable non-polar contributions are a significant factor in the stability of these interactions. Based on the analysis of the free energy landscapes, Mol-7112 is likely to form the most stable and specific interaction with the ASPM calponin domain. However, the flexibility observed in the interactions of Mol-7424 and Mol-273 could also be advantageous in therapeutic contexts, depending on the desired binding characteristics and the biological function of the ASPM calponin domain. In Table 1, ΔE_col describes the electrostatic interaction between ligand and receptor, and ΔE_cov describes the contribution from bonded interactions.

### 3.12 Evaluation of interaction stability using free energy landscape (FEL)

**FIGURE 6:**
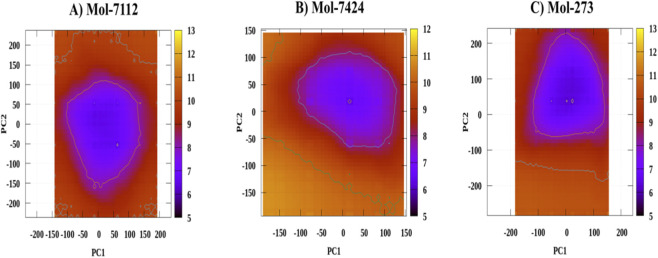
Free Energy Landscape of Phytochemical-CCNA2 Complexes. **(A)** Free energy landscape (FEL) plot for Mol-7112. **(B)** FEL plot for Mol-7424. **(C)** FEL plot for Mol-273. The FEL was generated using principal component analysis (PCA), where PC1 and PC2 represent the first two principal components of the system. The color gradient indicates the energy levels in kcal/mol, with purple representing the lowest energy regions and yellow representing the highest energy regions. The most stable conformations of the complexes are concentrated in the low-energy regions, illustrating the dominant conformational states sampled during the molecular dynamics simulation.

We further performed the free energy landscape to compare stability, specificity, and flexibility of interaction (Figure 6). Both Mol-7112 and Mol-7424 exhibit deep energy minima, which are indicative of stable interactions (Figures 6A–C). Mol-7112 appears to have a slightly smoother, more symmetrical basin, suggesting a more stable, specific binding mode compared to Mol-7424 (Figure 6A). The single, well-defined minimum for Mol-7112 suggests high interaction specificity, with fewer alternative conformations (Figure 6C). Mol-7424, while also showing a specific interaction, may allow for some alternative binding modes due to the ruggedness of the landscape. Mol-273’s broader energy basin suggests that this drug fragment may bind in multiple conformations, providing a more flexible interaction with the ASPM calponin domain (Figure 6B).

### 3.13 Computational evaluation of pharmacokinetic properties and toxicity risks

Using ADMET parameters, we observe that Mol-7424 meets Lipinski’s Rule of Five with zero violations and has a bioavailability score of 0.56, indicating drug-like properties with potential for good oral bioavailability (Supplementary Table S1). For comparative reasons, we also explored Mol-7112 (4,4′-dihydroxybiphenyl) and Mol-273 (1-BOC-3-piperidine carboxylic acid). Mol-7112 (MW = 186.21 Da, HBA = 2, HBD = 2, TPSA = 40.46 Å^2^, WLOGP = 2.76) is characterized by high GI absorption and BBB permeability, with a bioavailability score of 0.55 and no violations of Lipinski’s rule (Supplementary Figure S5). Similarly, Mol-273 (MW = 229.27 Da, HBA = 4, HBD = 1, TPSA = 66.84 Å^2^, WLOGP = 1.34) shows high GI absorption and BBB permeability, with an even higher bioavailability score of 0.85, and does not breach Lipinski’s rule. Such a comparison emphasizes a unique trait of Mol-7424, the lack of BBB permeability, which corroborates the selectivity toward peripheral tissues such as the liver.

Further, passive membrane permeability was evaluated using the SwissADME BOILED-Egg model to predict gastrointestinal (GI) absorption and blood-brain barrier (BBB) permeability (Supplementary Figure S5). Mol-7424 is predicted to exhibit high passive GI absorption, as corroborated by its low molecular weight and moderate polarity (TPSA = 77.76 Å^2^), thereby favoring oral administration. In contrast, poor BBB permeability is suggested, in line with its expected therapeutic function, targeting liver tissue rather than the CNS. Additionally, no P-glycoprotein (Pgp) substrate profile was attributed to Mol-7424, leading to reduced chances of efflux-mediated resistance affecting its pharmacokinetics. Besides, Mol-7424 has shown plasma protein binding (PPB) was 37.822 in ADMET lab analysis (Supplementary Material 1) with Clearance Rate (CLplasma): 4.75 mL/min/kg. It demonstrates that moderate plasma protein binding and acceptable clearance are favourable pharmacokinetic properties.

### 3.14 Evaluation of successful drug delivery via lipid bilayer environment

A molecular dynamics simulation of Mol-7424 with the lipid bilayer over 100 ns provides insights into the drug’s membrane permeability and interaction stability. The RMSD profile remained between 1.0 and 2.0 Å, indicating that Mol-7424 maintained conformational stability during its interaction with the membrane (Supplementary Figure S6A). Hydrogen bond analysis revealed up to four transient but consistent interactions throughout the trajectory, supporting the formation of stable contacts with lipid head groups (Supplementary Figure S6B). The minimum distance between the drug and lipid bilayer stayed consistently low (∼0.1–0.3 nm), reflecting sustained proximity and potential embedding within the bilayer (Supplementary Figure S6C). The penetration depth analysis confirmed a stable insertion of the drug into the membrane with an average depth of ∼2.5 nm, except for a brief fluctuation at ∼75 ns, which may represent a transient surface interaction (Supplementary Figure S6D). Furthermore, the radial distribution function (RDF) exhibited a sharp peak around 0.5 nm, signifying preferential spatial interaction between Mol-7424 and lipid atoms (Supplementary Figure S6E). Collectively, these findings indicate that Mol-7424 demonstrates strong membrane affinity, stable insertion, and favorable permeation dynamics, supporting its potential for efficient cellular uptake and drug delivery.

## 4 Discussion

ASPM’s involvement in the Wnt/β-catenin signaling pathway, a critical pathway in cancer progression, has been highlighted, suggesting that targeting ASPM could disrupt this pathway and inhibit tumor growth (Xu et al., 2021; Zou et al., 2019). Moreover, ASPM’s role in DNA repair mechanisms, particularly homologous recombination, has been demonstrated. ASPM interacts with BRCA1, protecting it from proteasomal degradation and ensuring efficient DNA repair (Xu et al., 2021). This function of ASPM in maintaining genome stability makes it a potential biomarker and key drug target in different cancer types, with its expression correlating with tumor grade and recurrence (Hagemann et al., 2008; Horvath et al., 2006; Zou et al., 2019). ASPM also plays a role in mitotic spindle organization, chromosome segregation, and maintenance of centrosome integrity, all of which are essential for cell cycle progression and survival in rapidly dividing cells (Higgins et al., 2010; Kouprina et al., 2005; Zhong et al., 2005). In glioblastoma, elevated ASPM expression is associated with malignant progression, promoting tumor growth by regulating G1 restriction point progression and Wnt-β-catenin signaling (Kato et al., 2011; Wu et al., 2022). This underscores ASPM’s multifaceted contributions to tumorigenesis across different cancer types.

This study highlights ASPM as a key biomarker in hepatocellular carcinoma (HCC), reinforcing previous reports that link its overexpression to poor prognosis and aggressive tumor behavior (Horvath et al., 2006; Zhang et al., 2021). The integration of multi-population transcriptomics ensures the robustness of these findings, as biomarker discovery across diverse cohorts enhances the generalizability and clinical relevance of the results (Williams et al., 2015). HCC exhibits substantial heterogeneity in its molecular landscape due to genetic, environmental, and lifestyle factors; thus, a comparative transcriptomic approach strengthens the reliability of identified targets by reducing population-specific biases (Tan et al., 2022).

Beyond biomarker identification, this study also advances therapeutic insights by targeting the ASPM calponin domain for drug discovery. The lack of structural data for ASPM previously hindered drug development efforts, but our computational modeling and docking analysis provide a framework for rational drug design. Among the identified small molecules, Mol-7424 exhibited the most stable interaction with ASPM, as supported by molecular dynamics simulations. This molecule demonstrated sustained hydrogen bonding and minimal RMSD fluctuations, indicating strong binding affinity and potential drug-like properties. Importantly, targeting ASPM’s calponin domain is a novel approach, as this region is implicated in cytoskeletal dynamics and mitotic integrity, making it an attractive target for disrupting tumor growth.

Mol-7424 (3,5-dihydroxybenzoic acid, 3,5-DHBA) emerged as the most promising compound in our study, exhibiting stable interactions with the ASPM calponin domain in both docking and molecular dynamics simulations. The hydroxyl and carboxyl functional groups in 3,5-DHBA are known to participate in strong hydrogen bonding and electrostatic interactions, features that have been observed in crystallographic and supramolecular studies of 3,5-dihydroxybenzoic acid derivatives, where networks of O–H…O hydrogen bonds play a key role in structural stability and intermolecular association (Burchell et al., 2001). 3,5-Dihydroxybenzoic acid has demonstrated potent competitive inhibition of tyrosine phenol-lyase *in vitro* and, upon oral administration, significantly reduced fecal phenol levels in mouse models, highlighting the ability of this phenolic scaffold to interact with protein targets and modulate biological pathways (Kobayashi et al., 2025). Additionally, recent studies developing tyrosinase inhibitors based on dihydroxyphenol structures highlight that hydroxylated benzene derivatives can modulate tyrosinase activity, an important consideration for potential off-target effects (Wang et al., 2025). These findings support our computational results, suggesting that the chemical features of 3,5-DHBA, particularly its hydroxyl and carboxyl groups, which are capable of forming stable hydrogen bonds with protein residues, may contribute to its favorable binding affinity for the ASPM calponin domain. Furthermore, these structural features provide opportunities for future chemical optimization to enhance solubility, potency, and selectivity during drug development. In contrast, future enzyme selectivity and toxicity studies will be essential to ensure the specificity and safety profile of Mol-7424 as an ASPM-targeted therapeutic agent.

Future work should prioritize experimental structural determination (e.g., cryo-EM) to refine target specificity. Second, while Mol-7424 demonstrates stable interactions in MD simulations, its therapeutic potential requires experimental validation, including CRISPR/Cas9-mediated ASPM knockdown to confirm on-target effects, co-immunoprecipitation assays to verify pathway disruption (e.g., Wnt/β-catenin or BRCA1 interactions), and *in vitro* cytotoxicity assays in HCC cell lines. Third, our screening was restricted to a 2,500-compound library; expanding to larger libraries (e.g., ZINC15) could yield candidates with enhanced pharmacokinetic properties. Finally, single-cell RNA-seq could resolve ASPM’s heterogeneity across HCC subtypes, and lipid bilayer-embedded MD simulations may better mimic *in vivo* membrane interactions. These steps will bridge computational insights to preclinical relevance, ensuring translational fidelity.

Future research should prioritize the experimental validation of Mol-7424 in preclinical HCC models to confirm its efficacy and safety. Given ASPM’s crucial role in Wnt/β-catenin signaling and homologous recombination-mediated DNA repair, inhibiting ASPM may disrupt tumor growth by impairing mitotic regulation and genome stability. The presence of hydroxyl groups in Mol-7424 provides opportunities for further chemical modifications to enhance its solubility, potency, and selectivity, critical factors in drug development. Additionally, exploring combination therapies with ASPM inhibitors and conventional treatments, such as chemotherapy or radiotherapy, could yield synergistic effects, potentially improving clinical outcomes in HCC patients. Beyond liver cancer, ASPM-targeted therapies may have broader applications across other malignancies where ASPM overexpression correlates with tumor aggressiveness and recurrence. Future studies will focus on extended simulations and experimental validation to further support the therapeutic potential of Mol-7424 in hepatocellular carcinoma.

### 4.1 Methodological limitations

While the approaches used in this study provide valuable insights into ASPM structure, dynamics, and ligand interactions, several limitations should be acknowledged. Blind docking may generate binding poses that do not fully correspond to the true binding site or accurately reflect binding energetics; therefore, docking results should be interpreted cautiously and considered as a guide for further experimental or computational validation. Additionally, longer MD simulations (e.g., 200–500 ns) may better capture rare conformational events. Finally, structural predictions and ligand interactions rely on modeled protein structures and may differ from *in vivo* conformations, which should be considered when interpreting the results.

## 5 Conclusion

In conclusion, this study underscores the importance of comparative transcriptomics in biomarker discovery, demonstrating how multi-population analyses can enhance the robustness and translational potential of findings. By integrating computational drug discovery with transcriptomic and structural biology approaches, this study provides a solid foundation for developing ASPM-targeted therapies. The identification of Mol-7424 as a promising ASPM inhibitor paves the way for future drug development efforts in HCC and potentially other cancers driven by ASPM overexpression.

## Data Availability

The original contributions presented in the study are included in the article/Supplementary Material, further inquiries can be directed to the corresponding author.

## References

[B1] Abraham M. J. Murtola T. Schulz R. Páll S. Smith J. C. Hess B. (2015). Gromacs: high performance molecular simulations through multi-level parallelism from laptops to supercomputers. SoftwareX 1–2, 19–25. 10.1016/j.softx.2015.06.001 1

[B2] Al-Khafaji K. Tok T. T. (2020). Molecular dynamics simulation, free energy landscape and binding free energy computations in exploration the anti-invasive activity of amygdalin against metastasis. Comput. Methods Programs Biomed. 195, 105660. 10.1016/j.cmpb.2020.105660 32726718

[B3] Aleksander S. A. Balhoff J. Carbon S. Cherry J. M. Drabkin H. J. Ebert D. (2023). The gene ontology knowledgebase in 2023. Genetics 224 (1), iyad031. 10.1093/genetics/iyad031 36866529 PMC10158837

[B47] Arain M. A. Khaskheli G. B. Barham G. S. Marghazani I. B. (2024). Lactoferrin's role in modulating NF-κB pathway to alleviate diabetes-associated inflammation: A novel in-silico study. Heliyon 10 (14), e34051. 10.1016/j.heliyon.2024.e34051 39092264 PMC11292243

[B4] Becht R. Kiełbowski K. Wasilewicz M. P. (2024). New opportunities in the systemic treatment of hepatocellular carcinoma—today and tomorrow. Int. J. Mol. Sci. 25 (3), 1456. 10.3390/ijms25031456 38338736 PMC10855889

[B5] Bell J. A. Cao Y. Gunn J. R. Day T. Gallicchio E. Zhou Z. (2012). PrimeX and the schrödinger computational chemistry suite of programs, 534–538.

[B49] Burchell C. J. Ferguson G. Lough A. J. Gregson R. M. Glidewell C. (2001). Hydrated salts of 3, 5-dihydroxybenzoic acid with organic diamines: hydrogen-bonded supramolecular structures in two and three dimensions. Structural Science 57 (3), 329–338. 10.1107/S0108768100019832 11373391

[B6] Bussi G. Donadio D. Parrinello M. (2007). Canonical sampling through velocity rescaling. J. Chem. Phys. 126 (1), 014101. 10.1063/1.2408420 17212484

[B7] Chin C. H. Chen S. H. Wu H. H. Ho C. W. Ko M. T. Lin C. Y. (2014). cytoHubba: identifying hub objects and sub-networks from complex interactome. BMC Syst. Biol. 8 (4), S11. 10.1186/1752-0509-8-S4-S11 25521941 PMC4290687

[B8] Dallakyan S. Olson A. J. (2015). Small-molecule library screening by docking with PyRx. Methods Mol. Biol. 1263, 243–250. 10.1007/978-1-4939-2269-7_19 25618350

[B9] Darden T. York D. Pedersen L. (1993). Particle mesh ewald: an N ⋅log(N) method for ewald sums in large systems. J. Chem. Phys. 98 (12), 10089–10092. 10.1063/1.464397

[B10] DennisG. ShermanB. T. HosackD. A. YangJ. GaoW. LaneH. C. (2003). DAVID: database for annotation, visualization, and integrated discovery. Genome Biol. 4 (5), P3. 10.1186/gb-2003-4-5-p3 12734009

[B11] Eberhardt J. Santos-Martins D. Tillack A. F. Forli S. (2021). AutoDock vina 1.2.0: new docking methods, expanded force field, and python bindings. J. Chem. Inf. Model. 61 (8), 3891–3898. 10.1021/acs.jcim.1c00203 34278794 PMC10683950

[B12] Fang Y. Liu W. Tang Z. Xiang J. Zhou Y. Song S. (2023). Monocarboxylate transporter 4 inhibition potentiates hepatocellular carcinoma immunotherapy through enhancing T cell infiltration and immune attack. Hepatology 77 (1), 109–123. 10.1002/hep.32348 35043976

[B13] Ganesan P. Kulik L. M. (2023). Hepatocellular carcinoma: new developments. Clin. Liver Dis. 27 (1), 85–102. 10.1016/j.cld.2022.08.004 36400469

[B14] Gao C. H. Yu G. Cai P. (2021). ggVennDiagram: an intuitive, Easy-to-Use, and highly customizable R package to generate venn diagram. Front. Genet. 12, 706907. 10.3389/fgene.2021.706907 34557218 PMC8452859

[B15] Garcia A. Mathew S. O. (2024). Racial/ethnic disparities and immunotherapeutic advances in the treatment of hepatocellular carcinoma. Cancers 16 (13), 2446. 10.3390/cancers16132446 39001508 PMC11240753

[B16] Gómez-Rubio V. (2017). Ggplot2 - elegant graphics for data analysis (2nd edition). J. Stat. Softw. 77, 1–3. 10.18637/jss.v077.b02

[B17] Hagemann C. Anacker J. Gerngras S. Kühnel S. Said H. M. Patel R. (2008). Expression analysis of the autosomal recessive primary microcephaly genes MCPH1 (microcephalin) and MCPH5 (ASPM, abnormal spindle-like, microcephaly associated) in human malignant gliomas. Oncol. Rep. 20 (2), 301–308. 10.3892/or_00000007 18636190

[B18] Hess B. Bekker H. Berendsen H. J. C. Fraaije J. G. E. M. (1997). LINCS: a linear constraint solver for molecular simulations. J. Comput. Chem. 18 (12), 1463–1472. 10.1002/(SICI)1096-987X(199709)18:12<1463::AID-JCC4>3.0.CO;2-H

[B19] Higgins J. Midgley C. Bergh A. M. Bell S. M. Askham J. M. Roberts E. (2010). Human ASPM participates in spindle organisation, Spindle orientation and cytokinesis. BMC Cell Biol. 11, 85. 10.1186/1471-2121-11-85 21044324 PMC2988714

[B20] Horvath S. Zhang B. Carlson M. Lu K. V. Zhu S. Felciano R. M. (2006). Analysis of oncogenic signaling networks in glioblastoma identifies ASPM as a molecular target. Proc. Natl. Acad. Sci. U. S. A. 103 (46), 17402–17407. 10.1073/pnas.0608396103 17090670 PMC1635024

[B21] Huang J. Mackerell A. D. (2013). CHARMM36 all-atom additive protein force field: validation based on comparison to NMR data. J. Comput. Chem. 34 (25), 2135–2145. 10.1002/jcc.23354 23832629 PMC3800559

[B23] Kato T. A. Okayasu R. Jeggo P. A. Fujimori A. (2011). ASPM influences DNA double-strand break repair and represents a potential target for radiotherapy. Int. J. Radiat. Biol. 87 (12), 1189–1195. 10.3109/09553002.2011.624152 21923303

[B24] Kobayashi T. Oishi S. Hara K. Matsui M. Mena P. Hashimoto H. (2025). 3,5-Dihydroxybenzoic acid as a potent inhibitor of tyrosine phenol-lyase decreases fecal phenol levels in mice. J. Med. Chem. 68 (8), 8786–8795. 10.1021/acs.jmedchem.5c00418 40173106

[B25] Kouprina N. Pavlicek A. Keith Collins N. Nakano M. Noskov V. N. Ohzeki J. I. (2005). The microcephaly ASPM gene is expressed in proliferating tissues and encodes for a mitotic spindle protein. Hum. Mol. Genet. 14 (15), 2155–2165. 10.1093/hmg/ddi220 15972725

[B26] Mark P. Nilsson L. (2001). Structure and dynamics of the TIP3P, SPC, and SPC/E water models at 298 K. J. Phys. Chem. A 105 (43), 9954–9960. 10.1021/jp003020w

[B29] Otasek D. Morris J. H. Bouças J. Pico A. R. Demchak B. (2019). Cytoscape automation: empowering workflow-based network analysis. Genome Biol. 20, 185. 10.1186/s13059-019-1758-4 31477170 PMC6717989

[B30] Páll S. Hess B. (2013). A flexible algorithm for calculating pair interactions on SIMD architectures. Comput. Phys. Commun. 184 (12), 2641–2650. 10.1016/j.cpc.2013.06.003

[B31] Pathak S. Sonbol M. B. (2021). Second-line treatment options for hepatocellular carcinoma: current landscape and future direction. J. Hepatocell. Carcinoma 8, 1147–1158. 10.2147/JHC.S268314 34584898 PMC8464222

[B32] Ritchie M. E. Phipson B. Wu D. Hu Y. Law C. W. Shi W. (2015). Limma powers differential expression analyses for RNA-sequencing and microarray studies. Nucleic Acids Res. 43 (7), e47. 10.1093/nar/gkv007 25605792 PMC4402510

[B48] Safdar M. Hassan F. Khan M. S. Khan A. H. Junejo Y. Ozaslan M. (2024). *In silico* analysis of polyphenols modulate bovine PPARγ to increase milk fat synthesis in dairy cattle via the MAPK signaling pathways. J. Anim. Sci. 102, skae248. 10.1093/jas/skae248 39210246 PMC11551727

[B33] Shaweta S. Akhil S. Utsav G. (2021). Molecular docking studies on the anti-fungal activity of Allium sativum (garlic) against mucormycosis (black fungus) by BIOVIA discovery studio visualizer 21.1.0.0. Ann. Antivirals Antiretrovir. 028–032, 28–32. 10.17352/aaa.000013

[B34] Sun H. Yang H. Mao Y. (2023). Personalized treatment for hepatocellular carcinoma in the era of targeted medicine and bioengineering. Front. Pharmacol. 14, 1150151. 10.3389/FPHAR.2023.1150151/BIBTEX 37214451 PMC10198383

[B35] Tan S. Chen W. Kong G. Lai W. (2022). ASPM may be related to the malignant progression of hepatitis B and is associated with a poor prognosis of hepatocellular carcinoma. Front. Bioinforma. 2, 871027. 10.3389/fbinf.2022.871027 36304312 PMC9580902

[B37] Vogel A. Meyer T. Sapisochin G. Salem R. Saborowski A. (2022). Hepatocellular carcinoma. Lancet 400 (10360), 1345–1362. 10.1016/S0140-6736(22)01200-4 36084663

[B50] Wang J. Sun Y. Jiang X. Wen H. Cai H. Feng M. (2025). Discovery of novel dihydroxyphenol tyrosinase inhibitors for treatment of pigmentation: from enzyme screening to three-dimensional human skin melanin evaluation. J. Medici Chem. 68 (22), 24047–24074. 10.1021/acs.jmedchem.5c01710 41222499

[B38] Webb B. Sali A. (2016). Comparative protein structure modeling using MODELLER. Curr. Protoc. Bioinforma. 2016, 5.6.1–5.6.37. 10.1002/cpbi.3 27322406 PMC5031415

[B39] Williams S. E. Garcia I. Crowther A. J. Li S. Stewart A. Liu H. (2015). Aspm sustains postnatal cerebellar neurogenesis and medulloblastoma growth in mice. Dev. Camb. 142 (22), 3921–3932. 10.1242/dev.124271 26450969 PMC4712878

[B40] Wu B. Hu C. Kong L. (2021). ASPM combined with KIF11 promotes the malignant progression of hepatocellular carcinoma *via* the Wnt/Β-catenin signaling pathway. Exp. Ther. Med. 22 (4), 1154. 10.3892/etm.2021.10588 34504599 PMC8393588

[B41] Wu X. Xu S. Wang P. Wang Z.-Q. Chen H. Xu X. (2022). ASPM promotes ATR-CHK1 activation and stabilizes stalled replication forks in response to replication stress. Proc. Natl. Acad. Sci. 119 (40), e2203783119. 10.1073/pnas.2203783119 36161901 PMC9546549

[B42] Xing Z. Chu C. Chen L. Kong X. (2016). The use of gene ontology terms and KEGG pathways for analysis and prediction of oncogenes. Biochimica Biophysica Acta (BBA) - General Subj. 1860 (11), 2725–2734. 10.1016/j.bbagen.2016.01.012 26801878

[B43] Xu S. Wu X. Wang P. Cao S.L. Peng B. Xu X. (2021). ASPM promotes homologous recombination-mediated DNA repair by safeguarding BRCA1 stability. iScience 24 (6), 102534. 10.1016/j.isci.2021.102534 34142045 PMC8184511

[B44] Zhang H. Yang X. Zhu L. Li Z. Zuo P. Wang P. (2021). ASPM promotes hepatocellular carcinoma progression by activating Wnt/β-Catenin signaling through antagonizing autophagy-mediated Dvl2 degradation. FEBS Open Bio 11 (10), 2784–2799. 10.1002/2211-5463.13278 34428354 PMC8487047

[B45] Zhong X. Liu L. Ailian Z. Pfeifer G. P. Xingzhi X. (2005). The abnormal spindle-like, microcephaly-associated (ASPM) gene encodes a centrosomal protein. Cell Cycle 4 (9), 1227–1229. 10.4161/cc.4.9.2029 16123590

[B46] Zou Y. F. Meng L. B. He Z. K. Chen H.H. Shan M. J. Wang D. Y. (2019). Screening and authentication of molecular markers in malignant glioblastoma based on gene expression profiles. Oncol. Lett. 18 (5), 4593–4604. 10.3892/ol.2019.10804 31611967 PMC6781560

