# Supplementary material for: Comparative transcriptomics and computational drug discovery identify ASPM as a key oncogenic driver and therapeutic target in hepatocellular carcinoma
Source: Front Bioinform. 2026 May 7;6:1795889. doi: 10.3389/fbinf.2026.1795889 (PMC13189905; doi:10.3389/fbinf.2026.1795889)
Supplement: Supplementary file 1 [file Supplementaryfile1.docx]

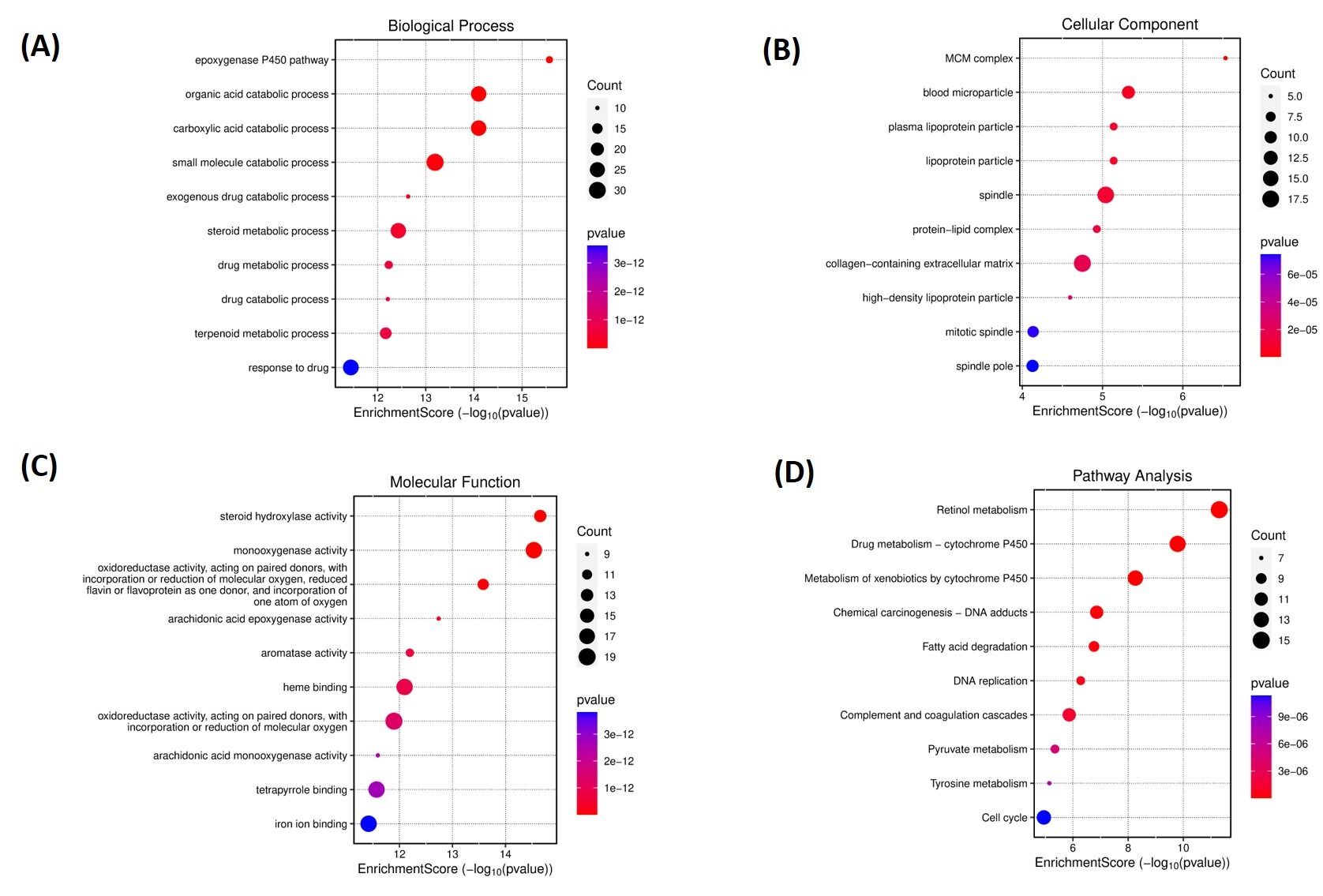


**Figure S1:** Bubble plots illustrating the enrichment scores and p-values for GO categories and KEGG pathways provide a visual representation of the critical functions and pathways associated with the identified DEGs (Figure S1). (A) GO in terms of biological processes (B) GO cellular components (C) GO in terms of molecular function (D) KEGG pathway analysis.


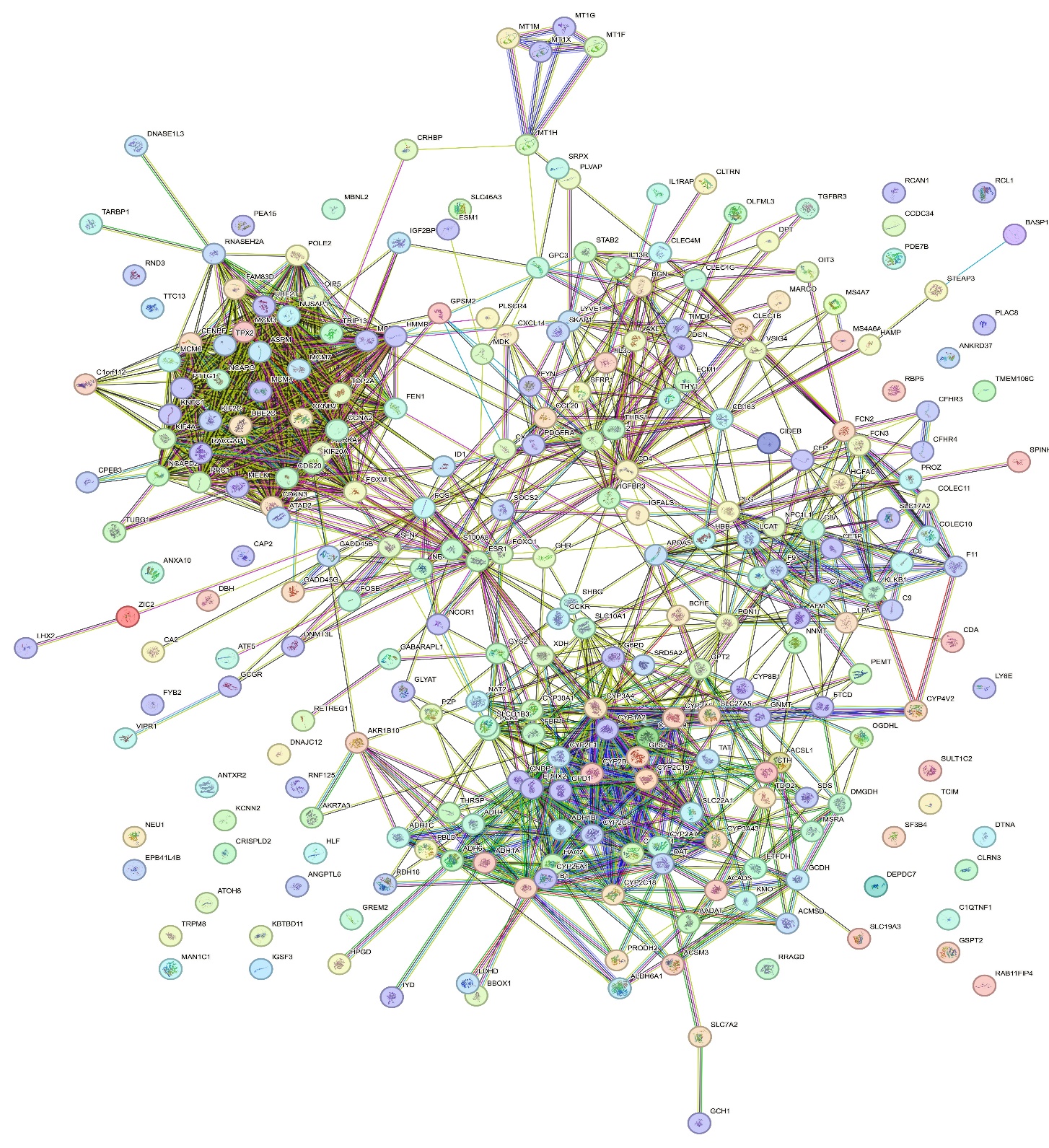


**Figure S2:** Kyoto Encyclopedia of Genes and Genomes pathway map illustrating differentially expressed genes (DEGs), as identified by protein-protein interaction network analysis using STRING. The network map was generated in the Database for Annotation, Visualization and Integrated Discovery 6.8. Each node is a differentially expressed gene. Network nodes represent proteins, edges represent protein protein associations

**Figure S3:** Evaluation of predicted calponin domain structure using Ramachandran plot via Molprobilty.


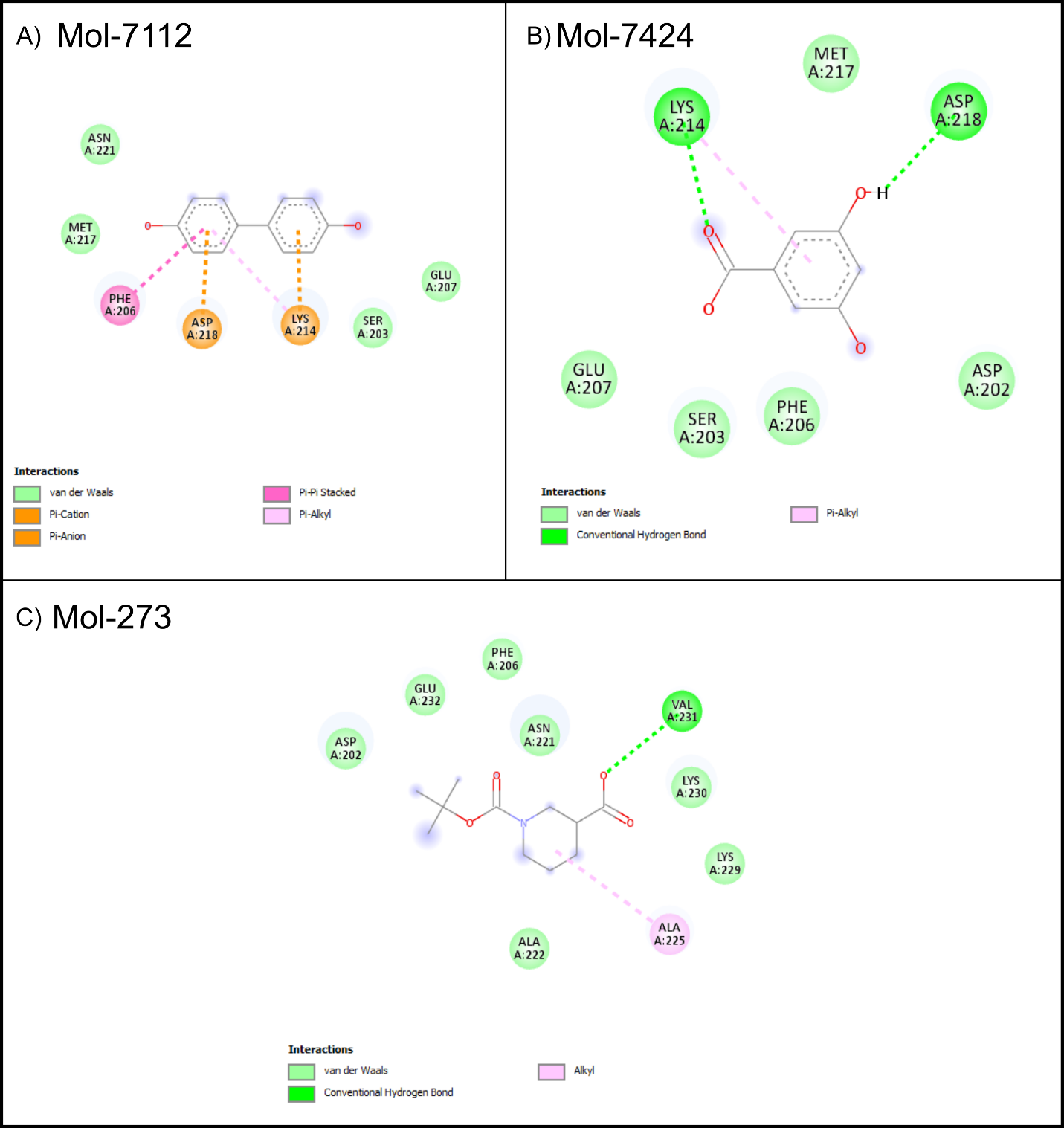


**Figure S4:** Two dimensional interaction analysis of ASPM protein with three drug fragments.


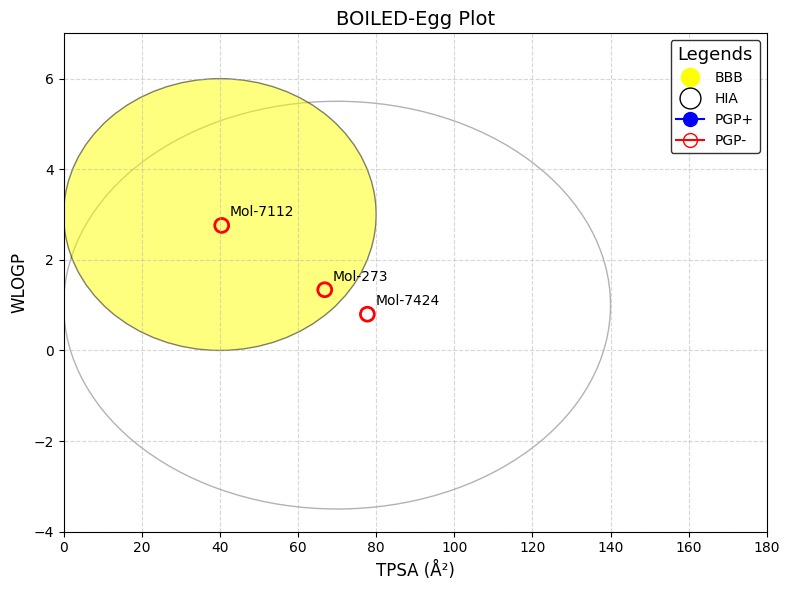


**Figure S5:** BOILED-Egg model to predict gastrointestinal (GI) absorption and blood-brain barrier (BBB) permeability


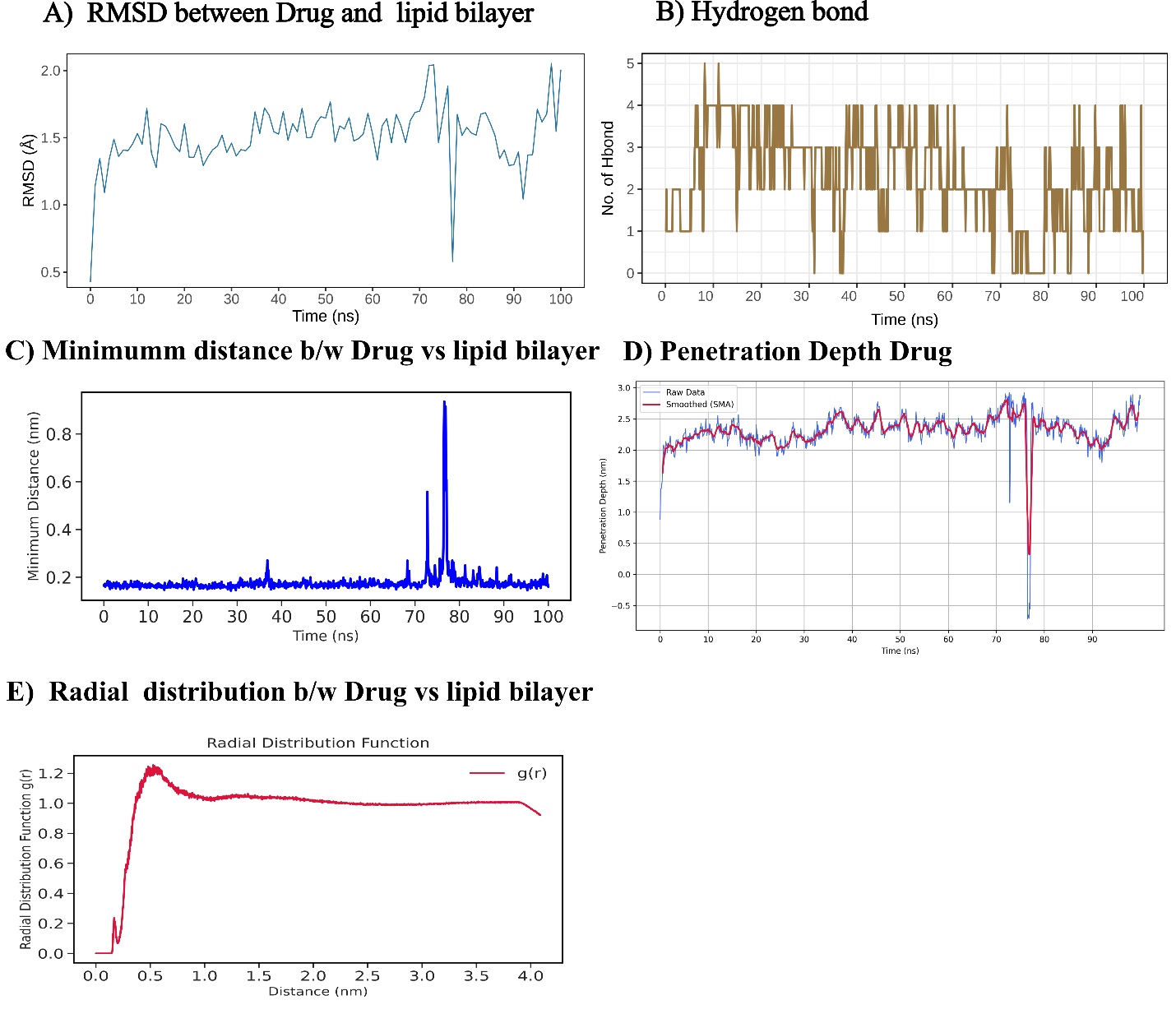


**Figure S6:** Molecular Dynamics Simulation Analysis of Drug-Lipid Bilayer Interactions.
